# Subtraction CT Angiography for the Evaluation of Lower Extremity Artery Disease with Severe Arterial Calcification

**DOI:** 10.3390/jcdd12040131

**Published:** 2025-04-02

**Authors:** Ryoichi Tanaka, Kunihiro Yoshioka

**Affiliations:** 1 Division of Dental Radiology, Department of Reconstructive Oral and Maxillofacial Surgery, Iwate Medical University, Iwate 020-8505, Japan; 2 Department of Radiology, Iwate Medical University, Iwate 028-3695, Japan; kyoshi@iwate-med.ac.jp

**Keywords:** CT/CTA, peripheral vascular disease, occlusion, stenosis/restenosis, arteriosclerosis, critical limb ischemia, subtraction

## Abstract

(1) Background: Peripheral arterial CT angiography (CTA) is an alternative to conventional angiography for diagnosing lower extremity artery disease (LEAD). However, severe arterial calcifications often hinder accurate assessment of arterial stenosis. This study evaluated the diagnostic performance of subtraction CTA with volume position matching compared to conventional CTA, using invasive digital subtraction angiography (DSA) as the gold standard. (2) Methods: Thirty-two patients with LEAD (mean age: 69.6 ± 10.8 years; M/F = 28:4) underwent subtraction CTA and DSA. The arterial tree was divided into 20 segments per patient, excluding segments with a history of bypass surgery. Subtraction was performed separately for each limb using volume position matching. Maximum intensity projections were reconstructed from both conventional and subtraction CTA data. Percent stenosis per arterial segment was measured using calipers and compared with DSA. Segments were classified as stenotic (>50% luminal narrowing) or not, with heavily calcified or stented segments assigned as incorrect. (3) Results: Of 640 segments, 636 were analyzed. Subtraction CTA and conventional CTA left 13 (2.0%) and 160 (25.2%) segments uninterpretable, respectively. Diagnostic accuracies (accuracy, precision, recall, macro F1 score) for subtraction CTA were 0.885, 0.884, 0.936, and 0.909, compared to 0.657, 0.744, 0.675, and 0.708 for conventional CTA. (4) Conclusions: Subtraction CTA with volume position matching is feasible and achieves high diagnostic accuracy in patients with severe calcific sclerosis.

## 1. Introduction

Lower limb arterial disease (LEAD) is an ischemic condition caused by arteriosclerosis. LEAD not only significantly impacts quality of life but can also be life-threatening, particularly in patients who develop critical limb ischemia (CLI) [1]. While diagnosing LEAD is relatively straightforward, detailed lesion characterization is crucial for selecting appropriate treatment options.

Contrast-enhanced CT angiography (CTA) is a non-invasive alternative to conventional angiography. Doppler ultrasound and magnetic resonance angiography (MRA) play a central role in imaging studies for diagnosing LEAD. Moreover, CTA can visualize luminal stenosis and vessel wall calcification, aiding in selecting invasive revascularization strategies, such as surgical bypass or vascular interventions [2]. It allows for precise grading of LEAD and detailed characterization of the vessel wall. However, CTA is limited in assessing luminal patency in severe arterial wall calcification [3,4].

Subtraction CTA is a promising alternative technique that may improve the visualization and characterization of arterial occlusive disease. Previous studies have demonstrated its effectiveness in diagnosing LEAD in the iliac artery [5]. More recently, its utility has been reported for the femoral artery and regions below the knee [6]. Understanding the state of blood flow disorders in the lower limbs has become increasingly important, especially in cases of severe ischemia, and advances such as dual-energy CT angiography have further strengthened diagnostic support [4].

On the other hand, in LEAD, comprehensive evaluation of the vascular tree of the entire lower limb is essential. Accurate evaluation of central and peripheral lesions simultaneously is crucial, as local evaluation alone is insufficient.

This study aims to evaluate the diagnostic performance of subtraction CTA compared with conventional CTA for assessing lower limb arterial stenosis in patients with severe arterial calcification. Using invasive digital subtraction angiography (DSA) as the gold standard, we evaluated the visualization ability of subtraction CTA from iliac artery lesions to below-the-knee artery lesions.

## 2. Materials and Methods

### 2.1. Study Design and Patient Population

This study was approved by our institute’s research ethical committee (approved No H23-105) and performed under the Declaration of Helsinki. All patients provided informed consent. Thirty-two consecutive LEAD patients who underwent CTA and DSA were prospectively included at our institution. All patients underwent clinically indicated subtraction CTA of the lower extremities for therapeutic planning. Diagnostic invasive DSA was performed before intervention. Accordingly, all subtraction CTA examinations were performed before DSA. The clinical characteristics of the study population are provided in Table 1.

### 2.2. CT Angiography

CT scanning was performed using a 64-slice scanner (Aquilion 64, Canon Medical Systems, Otawara, Japan) or a 320-slice scanner (Aquilion One, Canon Medical Systems, Otawara, Japan) in 32-slice mode with a slice thickness of 0.5 mm and a table feed of 13.5 mm/rot. The gantry rotation speed was 0.5 s/rot., the tube voltage was 120 kV, and the tube current was 75–125 mAs (variable tube current). Iterative reconstruction was applied during acquisition and reconstruction. Scanning was performed from the suprarenal abdominal aorta to the ankle joints. Automatic bolus tracking was used, with a threshold value of 170 HU set for a region of interest located in the abdominal aorta at the top of the scan range. After the acquisition of non-contrast images, 75 mL of Iopamidol (370 mgI/mL) or Iohexol (350 mgI/mL) was injected at 2 mL/s via an antecubital vein, followed by a 30 mL saline chaser.

**Figure 1:**
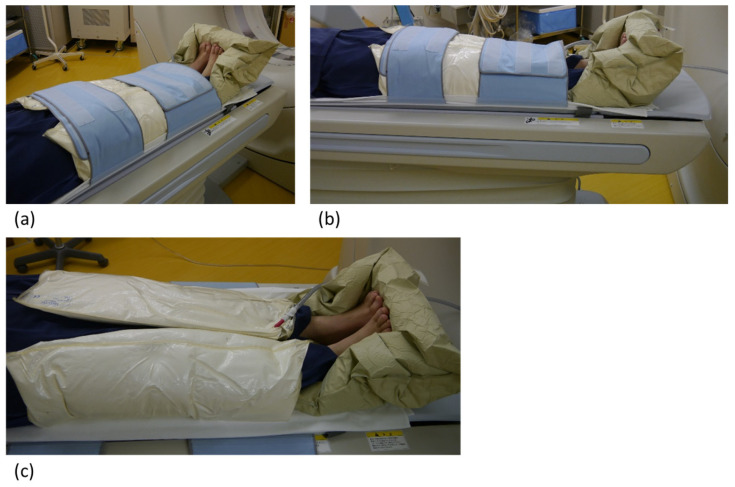
Patient immobilization system: Polystyrene bead bags were used for patient fixation. (**a**) and (**b**) illustrate the setup where the patient’s lower limbs, including the ankles and feet, are immobilized. The thighs and lower legs are secured to the bed using broad hook-and-loop straps. (**c**) When vacuumed, these bags harden, ensuring effective immobilization of the patient during the examination.

The subtraction CTA protocol consisted of a pre- and postcontrast acquisition [5,7]. Controlled orbit helical scanning [7,8,9] was performed to synchronize the gantry rotation and the table movement to obtain the same orbit of the helical row dataset for both image acquisitions and minimize the difference in helical artifacts. In addition, a patient immobilization system was used for reducing motion artifacts (Figure 1).

**Figure 2:**
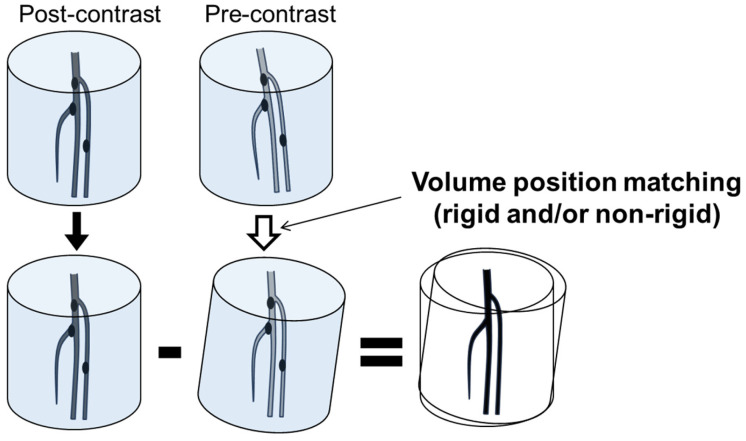
The basic concept of subtraction CTA with volume position matching.

**Figure 3:**
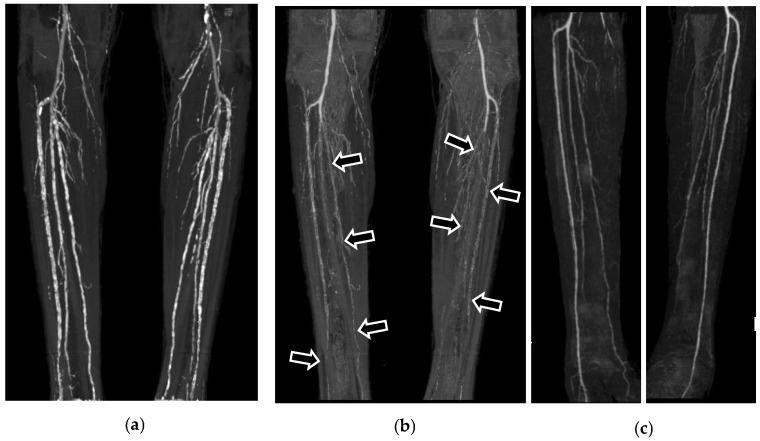
The difference between automatic and semi-automatic manipulation of subtraction CTA with volume position matching. (**a**) The non-subtracted MIP image shows diffuse arterial wall calcification. (**b**) The automatically subtracted CTA image, without manual manipulation, shows deletion of the vascular lumen due to misregistration of calcifications (black arrows). (**c**) The CTA image subtracted using a semi-automatic approach with manual adjustments accurately depicts the vascular lumen.

Images were reconstructed with a 1 mm slice thickness and a smooth reconstruction kernel (FC03). Subtraction was performed separately on the left and right limbs to achieve higher precision using the built-in application of the CT system. The volume position matching technique was used for the subtraction to minimize misregistration artifacts. The minimal unit of the position matching was 0.01 mm and 0.01 degrees for each X, Y, and Z volume axis. The position matching was performed with semi-automated, non-rigid registration by observing the reduction in artifacts. To avoid calculation errors, voxels with negative CT values were not subtracted. Negative CT values represent fat or air, and the subtraction of negative CT values could falsely result in high positive CT values in case of misregistration. Otherwise, intentional manipulation of CT values was not performed. Subsequently, the converted volume of non-enhanced CT data was subtracted from the volume of contrast-enhanced CT with the volume position matching technique (Figure 2 and Figure 3).

To minimize misregistration artifacts, subtraction images were calculated after aligning the vascular structures within the volume data.

**Figure 4:**
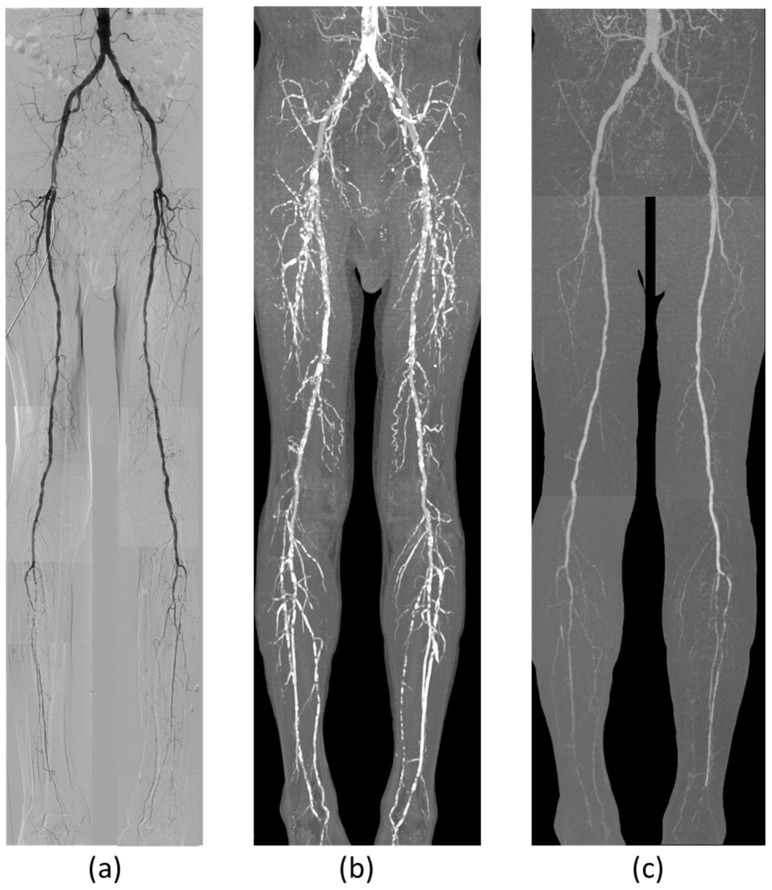
Visualization of DSA, conventional CTA, and subtraction CTA: (**a**) Composite image of bolus tracking DSA from the abdominal aorta to the ankle; (**b**) Composite MIP image of conventional CTA from the abdominal aorta to the ankle, showing clear visualization of arterial wall calcifications; however, evaluating luminal patency remains challenging; (**c**) Composite MIP image of subtraction CTA from the abdominal aorta to the ankle. Arterial wall calcifications are effectively eliminated, providing a clear visualization of luminal patency comparable to DSA.

All calculated data were reconstructed as maximum intensity projection (MIP) images (Figure 4). MIP images were reconstructed separately into three parts (aortoiliac, femoropopliteal, and below-the-knee regions) to take advantage of the spatial resolution. Conventional MIP images with non-subtracted data were reconstructed for comparison with subtraction CTA. All measurements were performed using MIP images because this study aims to provide angiographical images and assess their diagnostic accuracies.

### 2.3. Digital Subtraction Angiography

DSA was performed on all patients and used as a reference standard. Images were acquired using a digital angiography system (Philips Medical System, Allura Xper FD20/10, Best, The Netherlands). The “bolus chase” technique was used [10]. A 65–70 mL contrast medium (300 mgI/mL Iopamidol, Bayer Japan, Tokyo, Japan) was injected via a 4 Fr universal flush catheter (TEMPO4, Cardinal Health Japan, Tokyo, Japan) at 7 mL/s.

### 2.4. Image Analysis

**Figure 5:**
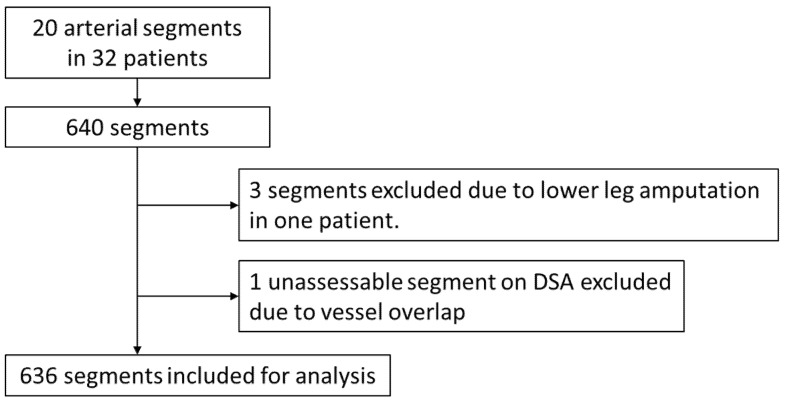
Flowchart of segment selection for evaluation.

Independent image analysis was performed on both CTA (conventional and subtraction) and DSA by a dedicated imaging clinical research organization (Micron Inc., Tokyo, Japan). Image evaluation was performed on a per-segment basis. The arterial tree was divided into 20 segments per patient, as follows: common iliac artery, external iliac artery, internal iliac artery, common femoral artery, superficial femoral artery, deep femoral artery, popliteal artery, anterior tibial artery, posterior tibial artery, and peroneal artery. One patient had undergone amputation of the right lower limb; therefore, three below-the-knee segments were not available. Also, one internal iliac artery segment was not evaluated on DSA due to overlapping by an external iliac artery; therefore, this one segment was also excluded. Segments were scored as assessable or not. Accordingly, 636 segments were available for evaluation (Figure 5).

The evaluation was performed by measuring the percent stenosis of the most severe lesion in each arterial segment using the caliper function. Segments were graded as either stenotic or not to calculate diagnostic accuracy. A stenotic lesion was defined as binary stenosis (>50%). Unassessable segments due to heavy calcifications or stent placement were assigned as missing values and treated as incorrect data during statistical calculations.

### 2.5. Statistical Analysis

Statistical analyses were performed using R version 4.4.2 [11] and the pROC package version 1.18.5 [12] for receiver operating characteristic (ROC) analysis. In the ROC analysis, missing values were treated as incorrect responses. With 2000 bootstrap replicates, the Venkatraman method was employed to compare the area under the curve (AUC) of two ROC curves.

The diagnostic accuracies (accuracy, precision, recall, and macro F1 score) of conventional and subtraction CTA were calculated against DSA. The correlation coefficients of conventional CTA and subtraction CTA with DSA were calculated using correlation analysis for the overall dataset. All statistical analyses were performed with a significance threshold of 0.05 and a confidence level of 95%.

## 3. Results

Table 1 shows patient characteristics. Briefly, 84.4% of patients had hypertension, while other risk factors were also prevalent. 31.3% of patients had chronic renal insufficiency, and 18.8% of patients underwent chronic hemodialysis. Seventy-eight percent of patients were Fontaine class II claudicants. Finally, CLI (defined as Fontaine class III or IV) was present in 18.8% of patients.

Iterative reconstruction (AIDR 3D) was used in the latter 20 patients, while the first 12 patients underwent CTA without iterative reconstruction. Details regarding the radiation dose of the entire examination are shown in Table 2. With iterative reconstruction, a reduced radiation dose of almost two-thirds was achieved while maintaining the image quality of CTA.

On conventional CTA, 160 arterial segments (25.2%) were unassessable (Table 3), with the majority (n = 152) being unassessable due to arterial calcification. In contrast, subtraction CTA rendered only thirteen arterial segments (2.0%) unassessable. In most instances, overlapping branches and misregistration of calcification interfered with the definitive evaluation of lesion severity.

Table 4 provides the correlations of percentage stenosis on conventional CTA and subtraction CTA with DSA. In quantifying lesion severity, subtraction CTA strongly correlated with DSA (R^2^ = 0.873). A weak correlation was observed between conventional CTA and DSA (R^2^ = 0.358), driven mainly by the high number of false positive segments due to calcifications. For both conventional and subtraction CTA, a discrepancy between stenosis on CTA and occlusion on DSA was observed for several segments.

The diagnostic accuracy and AUC of ROC analysis of conventional CTA compared to DSA were 0.657 and 0.594, respectively. For subtraction CTA, these values were 0.885 and 0.913, respectively. A comparison of AUC showed a significant difference (*p* < 0.05) between conventional CTA and subtraction CTA. In patients with critical limb ischemia, the diagnostic accuracy of subtraction CTA was 0.889, although that of conventional CTA was 0.513. Details are provided in Table 4.

## 4. Discussion

This study demonstrated that subtraction CTA was superior to conventional CTA in cases of LEAD with severe arterial wall calcification. It exhibited high overall diagnostic accuracy, especially in CLI, suggesting that subtraction CTA may be a viable alternative to invasive angiography.

Arterial wall calcification is a common manifestation of arteriosclerosis and can be categorized into two types: intimal calcification of atherosclerotic lesions and Mönckeberg-type medial calcification. The latter is commonly observed in patients with diabetes and end-stage renal disease (chronic renal failure) [13,14,15]. Critical limb ischemia frequently occurs in these patients, necessitating a detailed analysis of blood flow-limiting lesions to preserve the affected limbs. Furthermore, the prevalence and severity of vascular calcification are associated with cardiovascular disease and all-cause mortality in hemodialysis and peritoneal dialysis patients, providing critical prognostic information [16].

Multidetector CTA has been widely adopted for the initial diagnostic evaluation and treatment planning of patients with LEAD [17]. While CTA excels at detecting calcifications, the accurate assessment of arterial segments can be challenging due to “blooming artifacts” resulting from severe calcification.

Revascularization is essential not only for patients with claudication but also for patients with CLI to prevent tissue loss or avoid limb amputation [18]. However, determining the severity of the disease is crucial for establishing adequate inflow before restoring outflow, even in severely calcified arterial segments [19]. In this study, 18.8% of patients were on chronic hemodialysis because of renal insufficiency, and 18.8% of patients had CLI. Notably, half of the patients undergoing chronic dialysis also had CLI.

MRA has potential advantages in examining diffusely calcified vessels in patients with diabetes mellitus and chronic renal insufficiency. However, due to the risk of nephrogenic systemic fibrosis, contrast-enhanced MRA should be avoided in patients with chronic renal failure [20,21,22].

Although conventional CTA provides better spatial resolution than MRA, it has limitations in evaluating arterial segments with severe calcification [23].

Dual-energy CT angiography has been proposed for bone and plaque removal, and studies have shown promising correlations with invasive angiography [24]. However, dual-energy CT angiography’s accuracy in evaluating CLI calf arteries was insufficient. In addition, the specificity of dual-energy CT angiography was still significantly reduced in the presence of calcifications [25].

In this study, subtraction CTA successfully produced luminal images of lower extremity arteries comparable to those obtained through digital subtraction angiography, even for small peripheral arteries. Subtraction CTA effectively removed calcification and the associated blooming artifacts while preserving the structural detail of the lumen. However, structural misalignment between non-contrast and contrast-enhanced images—caused by respiratory motion, intestinal peristalsis, or involuntary leg movements—can generate artifacts and degrade image quality. We developed volumetric position matching software to address this and performed targeted image reconstruction for each limb. These enhancements minimized differences and achieved high spatial resolution. While the technique demands meticulous patient preparation, specialized scanning, and dedicated postprocessing, it facilitates improved image evaluation.

The main limitations of CTA include the need to administer iodinated contrast medium and high levels of radiation exposure [26]. In this study, the contrast volume was reduced to 70 mL. Although 60% of these patients were already undergoing dialysis, careful consideration is still required when deciding whether CTA is appropriate.

In one patient, insufficient contrast enhancement was observed. An additional scan of the below-knee arteries was performed immediately after the first contrast-enhanced scan; however, the contrast medium also failed to reach the ankle level in the second scan. The likely cause was increased microcirculation resistance due to severe arteriosclerosis, resulting in extremely slow arterial blood flow and an inability to complete the evaluation.

Subtraction CTA requires both non-contrast and contrast-enhanced images, leading to an increased radiation dose compared to a single contrast-enhanced CTA. However, non-contrast CT is necessary for LEAD patients to determine the extent and location of calcification for invasive revascularization. It is also helpful in distinguishing blooming and beam-hardening artifacts caused by severe calcification, making it a key component of the imaging protocol. Recent advances in iterative reconstruction techniques and AI-based image processing are expected to reduce radiation doses, although further evaluation is required substantially.

## 5. Conclusions

Subtraction CTA is a practical and clinically useful diagnostic tool in evaluating peripheral arterial occlusive disease with severe arterial wall calcification.

## Figures and Tables

**Table 1:** Patient characteristics (n = 32).

Characteristic	Value
Age (y)	
Mean ± SD	69.6 ± 10.8
Range	51–87
Gender (n)	
Male	28 (87.5%)
Female	4 (12.5%)
Body	
Length (cm)	160.8 ± 6.0
Weight (kg)	56.1 ± 10.4
Body mass index	21.7 ± 3.7
Risk factors (n)	
Hypertension	27 (84.4%)
Diabetes	10 (31.3%)
Hypercholesterolemia	13 (40.6%)
Smoking	8 (25%)
Previous smoking history	8 (25%)
Chronic renal failure	10 (31.3%)
Hemodialysis	6 (18.8%)
Fontaine classification (n)	
I	1 (3.1%)
IIa	8 (25%)
IIb	17 (53.1%)
III	2 (6.3%)
IV	4 (12.5%)
Duration between CTA and DSA (days)	
Mean ± SD	45.2 ± 22.0
Range	3–127

Values in parentheses are percentages. Abbreviations: SD: standard deviation, CTA: computed tomographic angiography, DSA: digital subtraction angiography.

**Table 2:** Radiation dose of subtraction CTA.

Metrics	Overall (mGy⋅cm)	Without IR (mGy⋅cm)	With IR (mGy⋅cm)
Mean ± SD	1707.5 ± 1050.4	2962.7 ± 117.8	977.7 ± 69.7
Range	589.0–3672.6	1456–3672	659–1826

Abbreviations: CTA: computed tomographic angiography, IR: iterative reconstruction, SD: standard deviation

**Table 3:** Characteristics of unassessable lesions on CT angiography.

Characteristic	Conventional CTA	Subtraction CTA
Total number of segments	636	
Unassessable segments	160 (25.2%)	13 (2.0%)
Calcification	152 (23.9%)	6 (0.9%)
Metallic stent	3 (0.5%)	0 (0.0%)
Overlap of branches	4 (0.6%)	5 (0.8%)
Unclear margin	1 (0.2%)	2 (0.3%)

Abbreviation: CTA: computed tomographic angiography.

**Table 4:** Diagnostic accuracy and ROC results of subtraction CTA and conventional CTA.

Region	Metrics	Conventional CTA	Subtraction CTA
Total	# of Missing Values	160 (25.16%)	13 (2.04%)
	Accuracy	0.657	0.885
	Precision	0.744	0.884
	Recall	0.675	0.936
	F1 Score	0.708	0.909
	Correlation with DSA (R^2^)	0.358	0.873
	AUC (95% CI) *	0.594 (0.546–0.642)	0.913 (0.886–0.940)
Iliac	# of Missing Values	98 (51.31%)	5 (2.62%)
	Accuracy	0.471	0.927
	Precision	0.741	0.971
	Recall	0.444	0.930
	F1 Score	0.555	0.950
	AUC (95% CI) *	0.343 (0.246–0.441)	0.933 (0.876–0.990)
Femoropopliteal	# of Missing Values	52 (20.31%)	6 (2.34%)
	Accuracy	0.707	0.891
	Precision	0.808	0.914
	Recall	0.768	0.934
	F1 Score	0.788	0.924
	AUC (95% CI) *	0.590 (0.500–0.680)	0.902 (0.851–0.952)
Below the Knee	# of Missing Values	10 (5.29%)	2 (1.06%)
	Accuracy	0.778	0.836
	Precision	0.633	0.689
	Recall	0.911	0.956
	F1 Score	0.747	0.808
	AUC (95% CI) *	0.830 (0.768–0.892)	0.903 (0.857–0.950)
CLI patients (6 cases, 111 segments)			
Total	# of Missing Values	49 (41.88%)	8 (6.84%)
	Accuracy	0.513	0.889
	Precision	0.571	0.892
	Recall	0.438	0.906
	F1 Score	0.496	0.899
	AUC (95% CI) *	0.443 (0.339–0.547)	0.854 (0.769–0.940)

* Indicates *p* < 0.05. Abbreviations: CTA: computed tomographic angiography, AUC: area under the curve, CI: confidence interval, CLI: critical limb ischemia

## Data Availability

The data presented in this study are available on request from the corresponding author due to ethical considerations (protection of sensitive personal information).

## Abbreviations

The following abbreviations are used in this manuscript:

CTA	computed tomography angiography
LEAD	lower extremity artery disease
CLI	critical limb ischemia
DSA	digital subtraction angiography
MRA	magnetic resonance angiography
MIP	maximum intensity projection
ROC	receiver operating characteristic
AUC	area under the curve
SD	standard deviation
CI	confidence interval

## References

[1] Aboyans V., Ricco J.B., Bartelink M.E.L., Bjorck M., Brodmann M., Cohnert T., Collet J.P., Czerny M., De Carlo M., Debus S. (2018). 2017 ESC Guidelines on the Diagnosis and Treatment of Peripheral Arterial Diseases, in collaboration with the European Society for Vascular Surgery (ESVS): Document covering atherosclerotic disease of extracranial carotid and vertebral, mesenteric, renal, upper and lower extremity arteriesEndorsed by: The European Stroke Organization (ESO) the Task Force for the Diagnosis and Treatment of Peripheral Arterial Diseases of the European Society of Cardiology (ESC) and of the European Society for Vascular Surgery (ESVS). Eur. Heart J..

[2] Itoga N.K., Kim T., Sailer A.M., Fleischmann D., Mell M.W. (2017). Lower extremity computed tomography angiography can help predict technical success of endovascular revascularization in the superficial femoral and popliteal artery. J. Vasc. Surg..

[3] Butt T., Lehti L., Apelqvist J., Gottsäter A., Acosta S. (2021). Influence of diabetes on diagnostic performance of computed tomography angiography of the calf arteries in acute limb ischemia. Acta Radiol..

[4] Koo B.-J., Won J.-H., Choi H.-C., Na J.-B., Kim J.-E., Park M.-J., Jo S.-H., Park H.-O., Lee C.-E., Kim M.-J. (2022). Automatic Plaque Removal Using Dual-Energy Computed Tomography Angiography: Diagnostic Accuracy and Utility in Patients with Peripheral Artery Disease. Medicina.

[5] Suzuki M., Tanaka R., Yoshioka K., Abiko A., Ehara S. (2015). Subtraction CT angiography for the diagnosis of iliac arterial steno-occlusive disease. Jpn. J. Radiol..

[6] Masuda T., Funama Y., Nakaura T., Sato T., Okimoto T., Masuda S., Yamashita Y., Yoshiura T., Noda N., Baba Y. (2021). Diagnostic performance of computed tomography digital subtraction angiography of the lower extremities during haemodialysis in patients with suspected peripheral artery disease. Radiography.

[7] Imakita S., Onishi Y., Hashimoto T., Motosugi S., Kuribayashi S., Takamiya M., Hashimoto N., Yamaguchi T., Sawada T. (1998). Subtraction ct angiography with controlled-orbit helical scanning for detection of intracranial aneurysms. AJNR Am. J. Neuroradiol..

[8] Nambu K., Suzuki R., Hirakawa K. (1995). Cerebral blood flow: Measurement with xenon-enhanced dynamic helical CT. Radiology.

[9] Watanabe Y., Kashiwagi N., Yamada N., Higashi M., Fukuda T., Morikawa S., Onishi Y., Iihara K., Miyamoto S., Naito H. (2008). Subtraction 3D CT Angiography with the Orbital Synchronized Helical Scan Technique for the Evaluation of Postoperative Cerebral Aneurysms Treated with Cobalt-Alloy Clips. Am. J. Neuroradiol..

[10] Jurriaans E., Wells I. (1993). Bolus chasing: A new technique in peripheral arteriography. Clin. Radiol..

[11] R Core Team (2024). R: A Language and Environment for Statistical Computing. R Foundation for Statistical Computing. https://www.R-project.org/.

[12] Robin X., Turck N., Hainard A., Tiberti N., Lisacek F., Sanchez J.-C., Müller M. (2011). pROC: An open-source package for R and S+ to analyze and compare ROC curves. BMC Bioinform..

[13] McCullough P.A. (2008). The Impact of Systemic Calcified Atherosclerosis in Patients With Chronic Kidney Disease. Adv. Chronic Kidney Dis..

[14] McCullough P.A., Agrawal V., Danielewicz E., Abela G.S. (2008). Accelerated atherosclerotic calcification and Monckeberg’s sclerosis: A continuum of advanced vascular pathology in chronic kidney disease. Clin. J. Am. Soc. Nephrol..

[15] Amann K. (2008). Media Calcification and Intima Calcification Are Distinct Entities in Chronic Kidney Disease. Clin. J. Am. Soc. Nephrol..

[16] Blacher J., Guerin A.P., Pannier B., Marchais S.J., London G.M. (2001). Arterial Calcifications, Arterial Stiffness, and Cardiovascular Risk in End-Stage Renal Disease. Hypertension.

[17] Heijenbrok-Kal M.H., Kock M.C.J.M., Hunink M.G.M. (2007). Lower Extremity Arterial Disease: Multidetector CT Angiography—Meta-Analysis. Radiology.

[18] Hiatt W.R. (2001). Medical Treatment of Peripheral Arterial Disease and Claudication. N. Engl. J. Med..

[19] Norgren L., Hiatt W.R., Dormandy J.A., Nehler M.R., Harris K.A., Fowkes F.G. (2007). Inter-Society Consensus for the Management of Peripheral Arterial Disease (TASC II). J. Vasc. Surg..

[20] Clorius S., Technau K., Watter T., Schwertfeger E., Fischer K.-G., Walz G., Gerke P. (2007). Nephrogenic systemic fibrosis following exposure to gadolinium-containing contrast agent. Clin. Nephrol..

[21] Thomaston J.L., Woldeyes R.A., Nakane T., Yamashita A., Tanaka T., Koiwai K., Brewster A.S., Barad B.A., Chen Y., Lemmin T. (2017). XFEL structures of the influenza M2 proton channel: Room temperature water networks and insights into proton conduction. Proc. Natl. Acad. Sci. USA.

[22] Malikova H., Holesta M. (2017). Gadolinium contrast agents—Are they really safe?. J. Vasc. Access..

[23] Ota H., Takase K., Igarashi K., Chiba Y., Haga K., Saito H., Takahashi S. (2004). MDCT Compared with Digital Subtraction Angiography for Assessment of Lower Extremity Arterial Occlusive Disease: Importance of Reviewing Cross-Sectional Images. Am. J. Roentgenol..

[24] Kau T., Eicher W., Reiterer C., Niedermayer M., Rabitsch E., Senft B., Hausegger K.A. (2011). Dual-energy CT angiography in peripheral arterial occlusive disease—Accuracy of maximum intensity projections in clinical routine and subgroup analysis. Eur. Radiol..

[25] Klink T., Wilhelm T., Roth C., Heverhagen J.T. (2017). Dual-Energy CTA in Patients with Symptomatic Peripheral Arterial Occlusive Disease: Study of Diagnostic Accuracy and Impeding Factors. Rofo-Fortschritte Geb. Rontgenstrahlen Bild. Verfahr..

[26] Jakobs T.F., Wintersperger B.J., Becker C.R. (2004). MDCT-imaging of peripheral arterial disease. Semin. Ultrasound CT MRI.

